# Response to Treatment with Botulinum Neurotoxin A (BoNT-A) in Children and Adolescents with Neurogenic Lower Urinary Tract Dysfunction and Idiopathic Overactive Bladder: A Systematic Review and Meta-Analysis

**DOI:** 10.3390/toxins16100443

**Published:** 2024-10-15

**Authors:** Andrea Panunzio, Rossella Orlando, Giovanni Mazzucato, Sonia Costantino, Giulia Marafioti Patuzzo, Clara Cerrato, Rita De Mitri, Vincenzo Pagliarulo, Alessandro Tafuri, Antonio Benito Porcaro, Alessandro Antonelli, Riccardo Giuseppe Bertolo, Luca Giacomello, Maria Angela Cerruto

**Affiliations:** 1 Department of Urology, “Vito Fazzi” Hospital, Piazza Filippo Muratore 1, 73100 Lecce, Italy; panunzioandrea@virgilio.it (A.P.); ritdemitri4@gmail.com (R.D.M.); enzopagliarulo@yahoo.com (V.P.); aletaf@hotmail.it (A.T.); 2 Urology Clinic, Department of Surgery, Dentistry, Paediatrics and Gynaecology, University of Verona, Azienda Ospedaliera Universitaria Integrata di Verona, Piazzale Aristide Stefani 1, 37126 Verona, Italy; orlandorossella92@gmail.com (R.O.); dott.giovannimazzucato@gmail.com (G.M.); soniacostantino1@gmail.com (S.C.); giulia.marafioti@studenti.univr.it (G.M.P.); drporcaro@yahoo.com (A.B.P.); alessandro.antonelli@univr.it (A.A.); riccardogiuseppe.bertolo@univr.it (R.G.B.); 3 University Hospital Southampton NHS Trust, Southampton SO16 6YD, UK; clara.cerrato01@gmail.com; 4 Paediatric Surgery Clinic, Department of Surgery, Dentistry, Paediatrics and Gynaecology, University of Verona, Azienda Ospedaliera Universitaria Integrata di Verona, Piazzale Aristide Stefani 1, 37126 Verona, Italy; luca.giacomello@univr.it

**Keywords:** clostridium botulinum, detrusor overactivity, neurogenic bladder, paediatric urology, transitional urology

## Abstract

Introduction: Botulinum neurotoxin A (BoNT-A) is a treatment option for neurogenic lower urinary tract dysfunctions (NLUTD) and idiopathic overactive bladder (OAB) in adults. Recently, its use has gained popularity in paediatric urology. Transitional urology deals with adolescents affected by congenital urological issues, who mature into adulthood. The aim of this systematic review was to update the current knowledge on the use of BoNT-A in children and adolescents. Methods: A comprehensive search in PubMed, Scopus, and Web of Science databases was performed from articles published up to September 2024. Both prospective and retrospective single-cohort or comparative studies evaluating outcomes of interest were included. These consisted of the amelioration of urinary incontinence (UI), continence rates, improvement of urodynamic parameters (maximum detrusor pressure during voiding, maximum bladder capacity, and bladder compliance), and type and prevalence of adverse/side effects. Qualitative and quantitative data syntheses were provided. Moderators and meta-regression analyses were carried out as well. Results: Forty-one full-text manuscripts were selected of which 26 focused on children with NLUTD, 13 on idiopathic OAB, and two on both conditions. Overall, 1521 patients were included of whom 715 were male, 646 female, and 160 of unknown sex. Mean age varied between 5.6 and 15.6 years. No studies specifically focused on transitional urology, despite patients up to at least 17 years of age being included. Several differences existed in design, type, dose, way of administration, outcomes measured and follow-up time; however, all studies independently showed an improvement of UI and urodynamic parameters with no major side/adverse events. Pooled analysis showed a mean rate of improvement in UI scores/episodes of 75.87% within a period of 3–6 months following BoNT-A treatment. Meta-regression analyses demonstrated a significant correlation between dryness rate and both patients’ age (negative) and bladder compliance (positive). Conclusions: Several uncontrolled or comparative studies provided significative evidence of the clinical benefit and safety of BoNT-A administration in children in terms of UI relief and improvement of urodynamic parameters, with neurogenic aetiologies being the most investigated conditions. A reduced bladder compliance was identified as one of the potential predictors of poor response to BoNT-A. Moreover, the earlier the treatment was started the higher the success rate that was reached in terms of dryness/urinary continence achievement.

## 1. Introduction

Botulinum neurotoxin A (BoNT-A) is a neurotoxin produced by *Clostridium botulinum*, which acts by blocking the exocytosis of acetylcholine on the presynaptic cholinergic nerve terminals among peripheral nerves, resulting in muscle relaxation, even in the bladder, and producing an analgesic effect [1]. In the adult population, BoNT-A is widely used to manage lower urinary tract dysfunctions (LUTD), including idiopathic overactive bladder (OAB), refractory to conservative treatments and first-line pharmacotherapy, and neurogenic detrusor overactivity [2,3,4], as recommended by international guidelines [5,6]. Additionally, its use is contemplated in the treatment of chronic pelvic pain of various origins, such as interstitial cystitis/bladder pain syndrome and chronic prostatitis/prostate pain syndrome [7]. 

In children, LUTD can be the result of disorders of the filling phase, the voiding phase, or a combination of both; underlying conditions may include OAB and dysfunctional voiding [8]. Epidemiological studies have shown that LUTD are common clinical disorders in the paediatric population with a prevalence ranging from 1 to 20%, and comprise up to 40% of outpatient paediatric urologist clinic visits [9,10]. The most common symptom is urinary incontinence (UI) for which in most cases there is not an obvious recognized cause; otherwise, UI may be explained by the presence of congenital anatomical or neurological abnormalities such as ectopic ureter, bladder exstrophy, or spina bifida/myelomeningocele [11]. The latter represents the most common neurological disorder responsible for bladder dysfunction in paediatric patients, with traumatic and neoplastic spinal cord lesions being less frequent [12,13]. According to international guidelines the treatment of LUTD in children is based on a multimodal approach such as behavioural modification, anticholinergic medication, and physiotherapy [11]. In the last two decades the use of BoNT-A has also gained popularity in paediatric urology due to evidence of its initial effectiveness coming from clinical studies focusing especially on children with neurogenic detrusor overactivity, and idiopathic detrusor overactivity with OAB symptoms, who were under intermittent catheterization and failed first-line anticholinergic therapy [14,15,16]. Indeed, in this subset of patients it is essential to prevent urinary tract deterioration and renal damage due to high bladder pressure and vesical–ureteral reflux, and to reach urinary continence with the less invasive treatment option, thus possibly improving quality of life (QoL) [17]. Transitional urology is the branch of urology which deals with adolescents affected by congenital urological issues, who mature into adulthood [18]. These subjects represent a subpopulation that is highly distinct from their adult and paediatric counterparts, who may require dedicated healthcare, in terms of proper management, adequate follow-up, and retreatment [18,19]. 

In 2009, DasGupta and Murphy published the results of the first systematic review on intravesical or intrasphincteric BoNT-A administration in children, discussing mechanisms of action, efficacy, and safety [20]. These authors reported an objective improvement in social continence and urodynamic parameters among included preliminary studies, but also highlighted the absence of placebo-controlled trials, as well as of longer-term data and rates of retreatment [20]. In the last 15 years, several additional studies have independently provided evidence for the clinical effectiveness of BoNT-A in the paediatric population with a negligible rate of side effects or complications [21,22]. However, currently, the use of sub urothelial or intradetrusorial injections of BoNT-A is still considered off-label by international guidelines in children with neurogenic LUTD (NLUTD) refractory to anticholinergics [11]. 

The aim of this systematic review was to provide a contemporary update on the use of BoNT-A in paediatric and transitional urology; specifically, we aimed to quantify its real and global benefit in terms of relief of lower urinary tract symptoms (LUTS), and an improvement of urodynamic parameters, in addition to identifying possible predictors of response and report type and prevalence of side effects, if any, among children and adolescents affected by NLUTD and idiopathic OAB. 

## 2. Results

**Figure 1:**
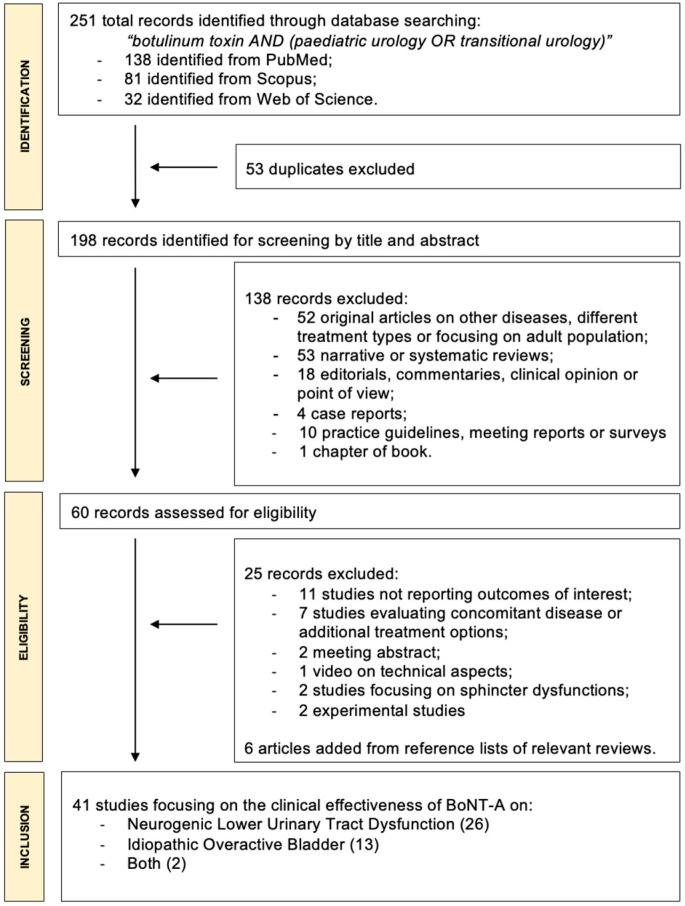
PRISMA (Preferred Reporting Items for Systematic Review and Meta-Analyses) flow diagram for the identification and selection of studies assessing the efficacy of botulinum neurotoxin A (BoNT-A) in paediatric and transitional urology.

The PRISMA diagram shows the literature research results (Figure 1). Overall, 251 records were identified; after excluding duplicates, 198 records remained for screening based on title and abstract. A total of 60 full-text manuscripts were then retrieved and assessed for eligibility, of which were excluded studies not reporting outcomes of interest, those evaluating concomitant disease or additional treatment options, those focusing on sphincter dysfunctions, experimental studies, a meeting abstract, and a video-article illustrating technical aspects of BoNT-A administration. Finally, from reference lists of the most relevant reviews, six additional articles were retrieved. Therefore, 41 full-text manuscripts met the inclusion criteria [14,15,23,24,25,26,27,28,29,30,31,32,33,34,35,36,37,38,39,40,41,42,43,44,45,46,47,48,49,50,51,52,53,54,55,56,57,58,59,60,61], and were considered for the present systematic review, of which 26 focused on the efficacy of BoNT-A in children affected by NLUTD [14,23,24,25,26,27,28,29,30,31,32,33,34,35,36,37,38,39,40,41,42,43,44,45,46,61], 13 evaluated BoNT-A in children with idiopathic OAB [15,47,48,49,50,51,52,53,54,55,56,57,60], and two tested BoNT-A in children affected by both conditions [58,59]. 

Overall, 1521 patients were included of whom 715 were male and 646 were female, with nine studies (n = 160 patients) not providing sex distribution among participants [30,32,37,38,39,41,42,55,59]. Mean age varied between 5.6 [27] and 15.6 [33] years. No studies specifically focused on the use of BoNT-A in transitional urology; however, adolescents up to at least 17 years were included in some studies [25,30,32,33,37,38,41,43,51,56,57,59]. Due to the significant heterogeneity in design, and outcomes measured by each study, to facilitate data comparison and interpretation, their main findings were separately reported according to the underlying condition to be treated and interventions that were compared. 

### 2.1. BoNT-A Administration in Patients with NLUTD

Twenty-six studies specifically investigated the role of BoNT-A in children with NLUTD and UI due to spina bifida/myelomeningocele, intraspinal astrocytoma, spinal cord injuries or trauma, and transverse myelitis [14,23,24,25,26,27,28,29,30,31,32,33,34,35,36,37,38,39,40,41,42,43,44,45,46,61]. Moreover, data on patients with neurogenic detrusor overactivity coming from two additional studies, which focused both on patients with NLUTD and idiopathic OAB [58,59] were also considered here. Of these studies, 13 were prospective [14,23,24,25,26,27,28,29,30,31,32,33,34], and 15 were retrospective [35,36,37,38,39,40,41,42,43,44,45,46,58,59,61]. Overall, participants included 806 children, with numbers ranging from 8 [38] to 113 [34], according to studies; they were distributed as follows according to sex: 359 males, 318 females, 129 of unknown sex. Mean age ranged between 5.6 [27] and 15.6 [33] years. 

Most studies reported that patients before to study participation had urodynamic proved detrusor overactivity with detrusor overactivity leak point pressure > 40 cmH_2_O, have failed, were intolerant, or experienced side/adverse events after previous conservative treatment as anticholinergic medication, and were under intermittent catheterization. The main contraindications consisted of urinary tract infections (UTI), coagulopathies, and allergy to BoNT-A, which represented exclusion criteria among considered studies. Patient assessment included medical history, physical and neurological examination, urinalysis and urine culture, abdominal ultrasound, urodynamic/video urodynamic study, bladder diary, and specific questionnaires on UI and QoL.

The type, dose, and way of administration of BoNT-A were variable, with 19 studies using onabotulinum toxin A (Botox^®^) [14,23,24,26,27,30,33,34,35,36,37,38,39,40,41,42,43,46,59], six studies using abobotulinum toxin A (Dysport^®^) [28,29,31,45,58,61], two studies testing both formulations [32,44], and one not specifying these data [25]. The dose administered mainly depended on body weight, ranging from 5 to 12 IU/kg up to a total of 300–500 IU according to formulations. Similarly, the number of intradetrusorial injections ranged between 10 and 50, mostly performed all over the bladder, sparing the dome and the trigone. Procedures were performed under general anaesthesia, using a rigid cystoscope, usually under antibiotic therapy.

Follow-up ranged between two weeks and 12 months. The main outcomes measured included urodynamic/uroflowmetry parameters, maximum bladder capacity (MBC), maximum detrusor pressure during voiding (PdetMax), bladder compliance (BC), incontinence episodes or score assessed through a dedicated questionnaire such as the daily incontinence score by Schurch [62] or the Arabic International Consultation on Incontinence Questionnaire—Urinary Incontinence Short Form, continence between intermittent catheterization, number UTI, patients’ satisfaction, clinical success, symptom improvement, or dryness rate.

The intervention and eventual control groups differed among included studies; 24 studies assessed the efficacy and safety of BoNT-A standard intradetrusorial injections [14,23,24,25,26,27,28,30,33,34,35,36,37,38,39,40,41,42,43,44,46,58,59,61]. All such studies were single-cohort trials, except for that of Safari et al., who compared BoNT-A intradetrusorial injections with and without additional injections in the external urethral sphincter [28], Austin et al., who separately tested 50 vs. 100 vs. 200 IU of Botox^®^ [34], Neel et al., who tested onabotulinum toxin A vs. its association with anticholinergics [27], and Hascoet et al., who tested onabotulinum toxin A vs. abobotulinum toxin A injections [44]. Conversely, in four studies BoNT-A was administered using the electro-motive drug administration (EMDA) delivery system [29,31,32,45] with controversial results. Although safe for most patients, Koh et al. [32] did not find any statistically significant improvement in any urodynamic parameter during the follow-up period. Conversely, both Ladi-Seyedian et al. [31] and Kajbafzadeh et al. [29] demonstrated a considerable and long-lasting improvement in UI and urodynamic parameters such as MBC and PdetMax. Moreover, when BoNT-A was administered through the EMDA delivery system a better sustained effect was observed compared to patients who received standard intradetrusorial injections [45].

### 2.2. BoNT-A Administration in Patients with Idiopathic OAB

Thirteen studies specifically investigated the role of BoNT-A in children with idiopathic OAB [15,47,48,49,50,51,52,53,54,55,56,57,60]. Moreover, data on patients with non-neurogenic detrusor overactivity coming from two additional studies, which focused both on NLUTD and idiopathic OAB [58,59], were also considered here. Of these studies, three were prospective [15,47,48], and 12 were retrospective [49,50,51,52,53,54,55,56,57,58,59,60]. Overall participants included 715 children, with numbers ranging from 4 [59] to 257 [56], according to studies, and including 356 males and 328 females (31 of unknown sex). Mean age ranged between 8.0 [56] and 12.3 [58] years. 

In each study, patients before enrolment had urodynamic proved detrusor overactivity that was inadequately managed by anticholinergic therapy. Exclusion criteria consisted of UTI, and evidence of significant dysfunctional voiding and post-void residual. Patient assessment included medical history, physical and neurological examination, urinalysis and urine culture, urodynamic study, bladder diary, and specific questionnaires on UI and QoL. 

Nine studies tested the efficacy of onabotulinum toxin A (Botox^®^) [51,52,53,54,55,56,57,59,60], five studies investigated the efficacy of abobotulinum toxin A (Dysport^®^) [15,48,49,50,58], and one did not specify the type of BoNT-A used [47]. The dose administered was 5 IU/kg up to a maximum of 100–500 UI according to formulations. Similarly, the number of injections ranged between 10 and 30, mostly performed all over the bladder, including the trigone. Follow-up ranged between one week and 12 months. The main outcomes measured included urodynamic/uroflowmetry parameters, incontinence episodes or score, patients’ satisfaction, clinical success, symptom improvement, or dryness rate.

The intervention and eventual control groups differed among included studies: 13 were single-cohort studies, which independently assessed the efficacy and safety of BoNT-A standard intradetrusorial injections [15,47,49,50,51,52,53,54,56,57,58,59,60]; conversely Ingham et al. compared the benefit of administering a high versus a low dose of BoNT-A [55], while Brown et al. [48] compared the effects of onabotulinum toxin A versus extended release tolterodine.

### 2.3. Pooled and Meta-Regression Analyses

Data from patients reporting the same outcomes at the same time point were pooled. A mean rate of improvement in UI of 75.87% (95% CI 67.87–83.87; *p* = 0.000; *I*^2^ 99.25%) was observed within a period of 3–6 months following BoNT-A treatment (23 studies) [14,15,25,26,27,29,30,31,32,33,34,37,42,43,44,46,47,49,50,52,53,57,58]. A mean rate of dryness of 51.24% (95%CI 36.51–65.97; random model with *p* = 0.000; *I*^2^ 99.84%) was observed within a period of 3–6 months following BoNT-A treatment (12 studies) [14,15,25,26,29,33,46,47,49,53,57,58]. Moderators’ analyses did not find statistically significant differences. The regression analysis demonstrated a statistically significant inverse correlation between dryness rate and patients’ age, indicating a progressive reduction in the former with increasing age (*p* = 0.026). Moreover, the regression analysis showed a statistically significant correlation between the dryness rate and the baseline BC (*p* = 0.001).

The pooled effect sizes of UI scores showed a significant reduction after BoNT-A treatment (Hedge’s *g* = −0.712; 95% CI from −0.87 to −0.55; *p* = 0.000; fixed model with *p* = 0.21; *I*^2^ 25.66%. Moderators’ analyses did not find statistically significant differences. The regression analysis demonstrated a statistically significant correlation between Hedge’s *g* and increasing PdetMax during voiding (*p* = 0.011) and an inverse correlation between Hedge’s *g* and increasing age.

The pooled effect sizes of PdetMax during voiding showed a significant reduction after BoNT-A treatment (Hedge’s *g* = −0.57; 95% CI from −0.66 to −0.48; *p* = 0.000; fixed model with *p* = 0.47; *I*^2^ 0%). Moderators’ analyses did not find statistically significant differences. The pooled effect sizes of BC showed a significant increase after BoNT-A treatment (Hedge’s *g* = 0.46; 95% CI from 0.30 to 0.62; *p* = 0.000; random model with *p* = 0.090; *I*^2^ 40.15%). Moderators’ analyses did not find statistically significant differences. The pooled effect sizes of MBC showed a significant increase after BoNT-A treatment (Hedge’s *g* = 0.59; 95% CI from 0.50 to 0.69; *p* = 0.000; fixed model with *p* = 0.90; *I*^2^ 0).

### 2.4. Type and Prevalence of Complications

Among the reported adverse/side events associated with BoNT-A, there is no mention of significant acute and chronic systemic toxicity [14,23,24,25,27,36,38,39,41,43,49,56,59], except from UTI [34,40,44,48,50,51,52,55,57,58,60], mild haematuria [33,47,53,57,58,60], and urinary retention [54,57] supporting its safety as an intravesical treatment. In BoNT-A administration through the EMDA delivery system, no serious side/adverse events were reported, except for transient skin erythema [29,32], and a burning sensation in the urethral orifice [29]. 

## 3. Discussion

This present comprehensive study has updated the current knowledge on the efficacy and safety of BoNT-A in children and adolescents with NLUTD or idiopathic OAB. We reported data on an overall cohort of 1521 patients (715 males, 646 females, 160 unknown sex) with a mean age ranging from 5.6 to 15.6 years, coming from 41 peer-reviewed publications up to September 2024. Of these, 26 and 13 deal with NLUTD and idiopathic OAB, respectively, and two with patients affected by both conditions. Several differences existed among included studies regarding the design, intervention and eventual control groups, sex and number of patients, age distribution, type, dose, and modality of BoNT-A administration, as well as outcomes measured and time of follow-up. However, the most investigated condition was detrusor overactivity due to neurogenic causes in children no longer responsive or unresponsive to conservative management and anticholinergic medications, who were under self-intermittent catheterization, with myelomeningocele being the most common underlying neurological disease. The most frequently used dose of BoNT-A was 10 IU/kg up to a maximum of 300–500 IU, depending on formulation, usually administered through 10–50 injections directly into the detrusor, sparing the trigone and using a rigid cystoscope under general anaesthesia. Finally, most studies independently confirmed the efficacy and safety of BoNT-A, showing an improvement in terms of dryness rate, UI episodes, and urodynamic parameters, with negligible side effects. 

These observations were also confirmed by the results of the pooled meta-analysis, where only cohorts of patients within included studies with available follow-up data on outcomes of interest were considered. Indeed, more than two thirds of patients, regardless of the type of bladder dysfunction, achieved an improvement in their urinary continence status, and at least one third were defined as dry. These results cannot be directly compared with other systematic reviews since pooled or meta-analysis have never been performed. However, Hoelscher et al. reported an estimated 33–69% decrease in UI episodes after BoNT-A injections among children with idiopathic OAB [63], while Zulli et al. reported a clinical success rate ranging from to 38 to 66% in children affected by NLUTD [64]. 

Evidence also suggests that the maximum benefit of BoNT-A is reached within 2 and 6 weeks, and that the effect is maintained up to 6–10 months [14,24,25], typically necessitating repeated injections, which can be challenging, especially in patients with neurological disease due to the repeated anaesthesia. Therefore, some authors reported their initial experience with BoNT-A administered through the EMDA delivery system with encouraging results. These considerations highlight the need for a strict follow-up with possible negative consequences on QoL. There is a little clear evidence of the real impact of BoNT-A on QoL of patients and parents [33,48]. Hui et al. reported a statistically significant improvement of children QoL 12 weeks after a BoNT-A injection compared to baseline [33], while Brown et al. showed that both patients and parents QoL increased after BoNT-A administration, with the effect lasting up to six months [48]. However, both contributions focused on NLUTD, and no study reported changes in QoL in idiopathic OAB. These findings may influence the decision to rely on concomitant or even alternative therapeutic options as augmentation cystoplasty. Recently, Li et al. investigated factors potentially associated, in children with NLUTD due to spina bifida, to the choice to undergo BoNT-A versus augmentation cystoplasty as the primary surgical treatment after failed medical management, and found that reduced MBC and a desire for independence/continence were statistically significantly associated with augmentation cystoplasty [65].

Understanding the prognostic factors or predictors of a poor response to BoNT-A is pivotal to properly managing and following up these patients. They have been well elucidated among adults and include type of bladder dysfunction and severity of the underlying neurological condition, patient age at the time of the starting treatment, history of previous treatment, baseline renal function, patient compliance and long-term adherence to the therapy, psychological factors influenced by family support or presence of comorbidities or frailty, in addition to various urodynamic variables as MBC, BC, and PdetMax [66,67,68,69]. Interestingly, results of the pooled analysis showed that the earlier the treatment was started, the higher the success rate that was reached in terms of dryness/urinary continence achievement. Similarly, lower PdetMax during voiding values, as well as higher BC values at the baseline, were associated with better dryness/urinary continence rates. Decreased BC is usually the sequela of neurologic disease but can also be the result of any process that destroys the elastic properties of the bladder, increasing the intravesical pressure, which can trigger the sensation of urgency and can precipitate the leakage of urine when the bladder pressure exceeds the urethral pressure [70]. Therefore, children with hypo compliant bladder are expected to be the least responsive to BoNT-A injections. These findings represent a novelty. Indeed, until now, no preoperative demographic or clinical factors have been identified as potential predictors of poor response to BoNT-A in children and adolescents. These considerations should be taken into account in order to select the optimal candidates for BoNT-A injections in order to achieve the best clinical outcome. 

Finally, most studies reported no acute or chronic major complications. Side effects, if present, were generally mild and included pain at the injection site, UTI and, in rare cases, urinary retention. However, it is always essential to monitor patients for possible complications, especially those with neurological comorbidities. 

Taken together, we can postulate that BoNT-A is an effective and well tolerated treatment option for children, especially those for which NLUTD is inadequately managed with anticholinergic therapy, producing results highly comparable to those of adults. However, response to treatment may vary and some patients may not achieve the expected benefits, thus necessitating ongoing assessment. Focusing on the clinical implications, and making a detailed analysis of all possible prognostic factors in the treatment of children and adolescents with NLUTD or idiopathic OAB using BoNT-A, may help to tailor treatment and improve patient outcomes.

The major limit of the present systematic review is the methodological heterogeneity among included studies. First, they differed in design and interventions; most were small-cohort studies accounting for fewer than 30 patients, and only 13 had a comparative arm, with four being randomized. Second, studies and patient numbers hugely varied according to underlying diseases and related grade of severity. Most focused on patients with NLUTD; therefore, conclusions could be drawn especially on this subset of patients. Despite the objective effectiveness demonstrated also in studies addressing idiopathic OAB, larger series on these conditions are required before recommended BoNT-A use as in adults. Third, patients’ demographics also varied according to studies; in this context, no study specifically focused on adolescent transitional patients. Young patients affected by LUTD, especially those suffering from neurogenic detrusor overactivity, will become adults and need proper management, adequate follow-up, and possible retreatment that should be guided by dedicated recommendations in order to prevent treatment discontinuation or an inadequate standard of care, which may lead to complications, thus worsening QoL [18,19]. Therefore, specific, dedicated, and well-designed investigations on this subpopulation should be encouraged. Fourth, there is lack of agreement on the type of BoNT-A to be used, as well as on the optimal dose, number, location of injections, and way of administration, which depend on disease aetiology and that remain to be addressed in adequately designed trials. BoNT-A formulation is an important issue to state when making a comparison because different types of BoNT-A showed different potency. Specifically, abobotulinum toxin is less potent compared with onabotulinum toxin [71]. Fifth, since follow-up time usually did not exceed 12 months, except for a few studies, data on long-term efficacy or timing to repeat injections were not available. Finally, despite no major concerns about adverse/side effects, most studies did not carefully describe type and prevalence of complications. The risk of urinary retention after treatment and the potential need for catheterisation should be considered and adequately reported; future contributions need to standardize the way of reporting complications and the time of their occurrence after injections. All these limitations should be considered when reading the results of the pooled analyses performed. 

## 4. Conclusions

The current systematic review represents the most contemporary and comprehensive update on BoNT-A in paediatric urology. In past years, several prospective and retrospective uncontrolled or comparative studies were designed, and provided additional evidence of the clinical benefit of BoNT-A in these patients, with detrusor overactivity due to neurological causes being the most investigated condition. To date, all published studies have shown an objective improvement in UI and urodynamic parameters, in addition to proving that BoNT-A administration is a feasible and safe procedure with no major side/adverse events. For the first time some baseline demographic (age) and urodynamic (low BC) variables have been identified as possible predictors of poor response to BoNT-A in paediatrics. Interestingly, meta-regression analysis showed that the earlier the treatment was started the higher the success rate that was reached in terms of dryness/urinary continence achievement. However, these findings will require additional investigations that should cover all the previously highlighted limitations to prove a clinically unquestionable effect of BoNT-A and justify its use and its acceptance as a part of the LUTD treatment algorithm.

## 5. Materials and Methods

### 5.1. Search Strategy, Selection of Eligible Studies, and Data Extraction

This systematic review was performed following the Preferred Reporting Items for Systematic Review and Meta-analyses (PRISMA) statement [72]. PubMed, Scopus, and Web of Science were searched systematically for articles published up to September 2024 investigating the clinical effectiveness of BoNT-A in paediatric and transitional urology. The key terms used for the search were as follows: botulinum toxin AND (paediatric urology OR transitional urology). The present study was registered with PROSPERO (International Prospective Register of Systematic Reviews) under the registration code CRD42024544998. 

Two paired investigators (A.P. and R.O.) independently screened all titles gathered from the literature review to identify potential eligible studies, and then evaluated full-text manuscripts to determine the final included ones (Figure 1). Any disagreements between the two independent authors were resolved by a third independent author (M.A.C.), after a collegial discussion to reach a final consensus. Prospective trials and retrospective analysis (single arm or comparative studies), with no limitation on patient number, with a minimum follow-up of one month, and reporting outcomes of interest, were included. These consisted of the amelioration of UI, and other LUTS if present, improvement of urodynamic parameters, and adverse/side effects. Meeting abstracts and case reports were excluded; articles focused on BoNT-A administration in adults, as well as on other diseases or treatment types, in-vivo experiments, editorial commentaries, chapters of book, practice guidelines, surveys, narrative or systematic reviews, and studies focusing on sphincter dyssynergia were also excluded. Reference lists of relevant and recent systematic reviews were also manually reviewed to identify potential supplementary studies of interest. The intervention whose efficacy was to be assessed was BoNT-A administration in children and adolescents during their transition toward the adult age for the treatment of NLUTD and idiopathic OAB. 

All data extracted from the included studies were recorded in an electronic database by two investigators (A.P. and R.O.). Collected data included main Author, journal, and year of publication; country of origin; design of the study; inclusion and exclusion criteria; recruitment period; intervention; type, dose, number, and location of BoNT-A injection/administration; number, age, and sex of participants; outcomes measured; follow-up time; and adverse events. Discrepancies, if any, were resolved by discussion between the two investigators until a consensus was reached or involving a third independent author (M.A.C.).

The primary outcomes were the amelioration of UI assessed through the evaluation of the number of episodes per days or with dedicated scores or questionnaires, and the improvement of urodynamic parameters, as PdetMax during voiding, BC, and MBC. The secondary outcome was the type and prevalence of the adverse/side effects.

### 5.2. Statistical Analyses

Data were synthesized using meta-analytic methods [73,74]. The standard mean difference, or the effect size between groups pre- and post- treatment, was calculated using Hedges’ *g* unbiased approach. Calculation of the effect sizes was based on means, differences in means, *p* values, and simple sizes of the analysed groups. Data were statistically pooled by the standard meta-analysis approach, meaning that studies were weighted by the inverse of the sampling variance. A test of heterogeneity was applied and the *I*^2^ statistic computed. The *I*^2^ statistic indicates the proportion of total variation among the effect estimates attributed to heterogeneity rather than sampling error and has the advantage to being intrinsically independent of the number of the studies. When the test of heterogeneity was not significant (*p* > 0.05) and/or *I*^2^ was less than 30%, a fixed model was adopted for evaluation of the results; otherwise, a random-effects model was used. Several characteristics within the analysed patients’ groups were identified and their effects on outcomes were examined. Categorical characteristics were treated as moderators and effectiveness was compared across subgroups by these moderators. Continuous characteristics were examined as covariates using random-effect (method of moments) meta-regression. We also assessed publication bias using Egger’s t test and funnel plots with a significance value based on 1-tailed *p* values [75,76]. Comprehensive Meta-Analysis V.4© software was used for statistical analyses. The statistics reported in this meta-analysis conformed to the PRISMA statement [72]. 

## Data Availability

All data supporting the reported results are available in the original studies included in the systematic review and were showed in figures and tables included in the present manuscript.
